# Failure Mechanisms of GFRP Scarf Joints under Tensile Load

**DOI:** 10.3390/ma14071806

**Published:** 2021-04-06

**Authors:** Carineh Ghafafian, Bartosz Popiela, Volker Trappe

**Affiliations:** Federal Institute for Materials Research and Testing (BAM), Unter den Eichen 87, 12205 Berlin, Germany; carineh.ghafafian@bam.de (C.G.); bpopiela999@gmail.com (B.P.)

**Keywords:** scarf joint, glass fiber reinforced polymers, failure mechanisms

## Abstract

A potential repair alternative to restoring the mechanical properties of lightweight fiber-reinforced polymer (FRP) structures is to locally patch these areas with scarf joints. The effects of such repair methods on the structural integrity, however, are still largely unknown. In this paper, the mechanical property restoration, failure mechanism, and influence of fiber orientation mismatch between parent and repair materials of 1:50 scarf joints are studied on monolithic glass fiber-reinforced polymer (GFRP) specimens under tensile load. Two different parent orientations of [−45/+45]_2S_ and [0/90]_2S_ are exemplarily examined, and control specimens are taken as a baseline for the tensile strength and stiffness property recovery assessment. Using a layer-wise stress analysis with finite element simulations conducted with ANSYS Composite PrepPost to support the experimental investigation, the fiber orientation with respect to load direction is shown to affect the critical regions and thereby failure mechanism of the scarf joint specimens.

## 1. Introduction

Fiber-reinforced polymer (FRP) composites are increasingly favored in the automotive, construction, aerospace, and wind industry due to their good strength- and stiffness-to-weight properties. With an increase in the use of these materials comes a rise in the necessity for their maintenance in service. This is especially significant in the wind turbine blade industry, where glass fiber-reinforced polymers (GFRP) are a key component. Here, imperfections during the manufacturing process lead to 70–80% of defects during service and thereby premature failure long before the projected 20-year design lifespan [1,2]. As an alternative to replacing entire turbine blades, localized repairs can be executed in the field by technicians accessing the blades directly by suspended roping. As opposed to full blade replacement, which leads to standstill and is an important inhibitor of turbine production, repairs on the ropes allow for a quicker return to service, thereby reducing potential loss of earnings. There is, however, no standardized practice for the localized repair of wind turbine blade shells, and methods often vary by manufacturer and turbine type. There is also a lack of understanding of the effects of various repair methods on the restored properties and endurance of the blade structure [3].

**Figure 1:**
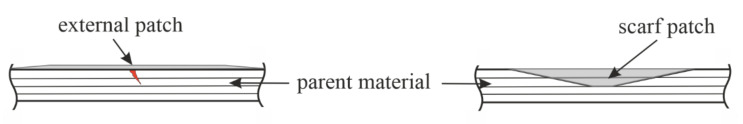
Two basic types of repair patches: external (**left**) and scarf (**right**). In both illustrations, the repair material is shown in gray, placed onto the white-depicted parent material, with an assumed damage shown in red.

Localized repair patches are nonetheless common practice in wind turbine blade repairs, as they are often utilized in the reinforcement and maintenance of advanced composite structures [4,5,6]. By creating a bond in a thin layer over a large joint area, they allow for more uniform stress distribution of the applied loads, leading to numerous advantages including better fatigue life, corrosion resistance, and compatibility with lightweight applications [7]. Two commonly used types of repair patches in composite materials are the external and scarf patch, illustrated in Figure 1. Both structures are depicted without an adhesive layer between, mirroring the methodology followed in this study.

Scarf repairs (Figure 1, right) are favored as the most efficient of the common structural joints [8,9]. They involve the removal of the damaged area with angled walls and the placement of new repair layers correspondingly on top. This leads to a nearly uniform shear stress distribution in the bond surface. Additionally, the angled joint walls allow for no eccentricity in the load distribution compared to joints with sharp corners, and thus lead to low peel stresses, which are a critical limiting component in adhesive joints [10,11,12]. The numerous advantages as well as the minor aerodynamic contour changes made possible by the smooth surface restoration make scarf repairs suitable for external structures [13].

**Figure 2:**
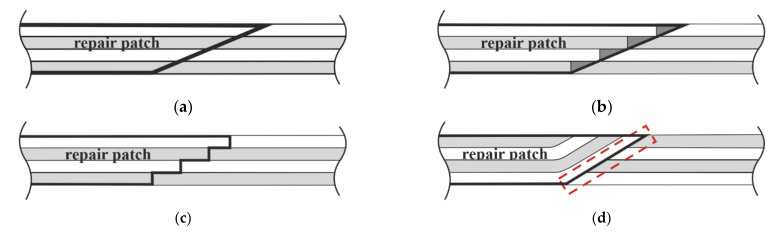
Possible scarf repair methods. Schematics represent the side cross-section of a scarf joint of a cross-ply monolithic laminate, with parent material on the left and repair material shown on the right of each illustration: (**a**) small-to-large for two hard structures being joined, (**b**) small-to-large with angled parent material walls, (**c**) modified small-to-large with stepped parent walls, and (**d**) large-to-small, with transition region of fiber orientation mismatch boxed in a dashed frame.

With their increase in use in lightweight application repairs, studies have been conducted to understand the effect of various scarf joint repairs on the overall mechanical properties. It has been shown that a decrease in scarf angle as well as an increase in external reinforcement plies on the surface of a repair leads to an increase in failure loads [14,15,16,17,18,19]. In practice and in the literature, a scarf ratio of 1:50 has been shown to be a good compromise between repair size and properties, with 70–80% static tensile strength restoration and over 80% post-impact residual tensile strength [3,12,20]. As a scarf patch carries the load of a post-repaired component, it has been shown to be most effective when the repair and parent ply stacking sequence are equivalent. Studies have examined small-to-large as well as a mix of large-to-small layups with different scarf ratio and found resin fillets to be important in controlling failure modes [21]. The difference between small-to-large and large-to-small layups is illustrated and explained in detail later in Figure 2. Non-traditional stacking sequences have also been studied under tensile in an effort to optimize the scarf repair patches, and strength restorations of over 85% have been calculated [22].

Additionally, the significance of the fiber orientation with respect to load direction between parent and repair material in scarf joint is not fully understood at present, and most studies have been conducted with CFRP materials. Depending on manufacturer and location on the blade structure, wind turbine blade shells are produced with different layup sequences. The influence of the stacking sequence on the laminate strength has been shown to be significant [17,23,24]. The interlaminar normal stresses are specifically important in discussing the effect of the stacking sequence [23]. Material discontinuities and elastic property mismatch in adjacent layers, as is the case for scarf joint repairs, lead to interlaminar stresses and thereby failure at loads much lower than predicted by the classical laminate theory. A variation in the interlaminar normal stresses through the thickness of symmetric cross-ply [90/0]_s_ graphite/epoxy circular cylindrical shell panels under uniform axial strain have been found near edges. The distribution of the interlaminar normal stresses have been found to be symmetric to the middle of the panel in the thickness axis [25]. Additionally, there exist sharp jumps in the interlaminar stresses between layers with different orientation [25,26,27].

Figure 2 shows scarf repair method application possibilities as a schematic, where light gray/white colors alternate to represent different fiber orientations. Figure 2a shows the ideal in terms of fiber orientation matching but is only possible for two hard materials being joined, for instance, plywood. Repairs are commonly executed with wet laminates laid onto a hard parent material in practice, as it is often impractical as a technician on the ropes to prepare repair patches in rigid form to exactly fit the geometry at hand. When performed with the small-to-large layup arrangement, shown in Figure 2b, resin pockets between the patch and parent material could appear as a consequence, illustrated with darker gray. This can compromise the overall component’s stiffness and strength. Siener’s experimental results of small-to-large CFRP scarf joints showed cracks consistently initiating and propagating along the patch side of the joint. This is because the stair-like interface of the patch bonding surface made it ill-suited for to bonding, and crack propagation occurred along the path with the least energy required for fracture, namely, on the side of the patch with a poor bonding surface [11]. A modified scarf method with a stepped transition zone, shown in Figure 2c, is an alternative to the small-to-large method commonly used in aircraft repairs which allows for direct matching of repair to parent plies. Its required level of precision, however, makes it impractical for rotor blade repairs to attain outside of controlled laboratory conditions. A large-to-small layup method, illustrated in Figure 2d, therefore often serves in practice as a compromise of practicality and restoration of mechanical properties, especially in the general aviation industry [28]. Here, however, since the first repair layer blankets the entire patch cutout and thereby all parent layers, a difference in fiber orientation in the transition region between parent material and patch leads to interlaminar shear stresses that could lead to early failure of the structure. Since a larger mismatch has been shown to lead to larger stresses [26], this region is of greater interest to study.

In a large-to-small scarf repair patch, there is to a certain extent a fiber orientation mismatch depending on the stacking sequence between the bottom-most patch layer on the parent layers, highlighted in Figure 2d. This transition region can lead to high interlaminar stresses, which could thus affect the failure mechanism. The role of the fiber orientation mismatch on the failure mechanism of a wet-to-hard structural scarf joint with no additional adhesive layer, especially for GFRPs, is thereby the motivation in this study. The aim of this paper is to determine the critical regions as well as the method of damage in scarf joints under static tensile load and thus provide insights to the role of the fiber orientation mismatch so as to be able to ultimately better design and utilize such joints in wind turbine blade shell repairs. This is carried out experimentally with monolithic GFRP scarf joint coupon specimens, and detailed insights are qualitatively enhanced with a layer-wise stress analysis using finite element analysis (FEA). The findings in this study can provide insight for the further improvement of scarf joint repairs by better understanding the role of the fiber orientation on the failure mechanism in these primarily GFRP structures.

## 2. Materials and Methods

### 2.1. Experimental Setup

**Figure 3:**
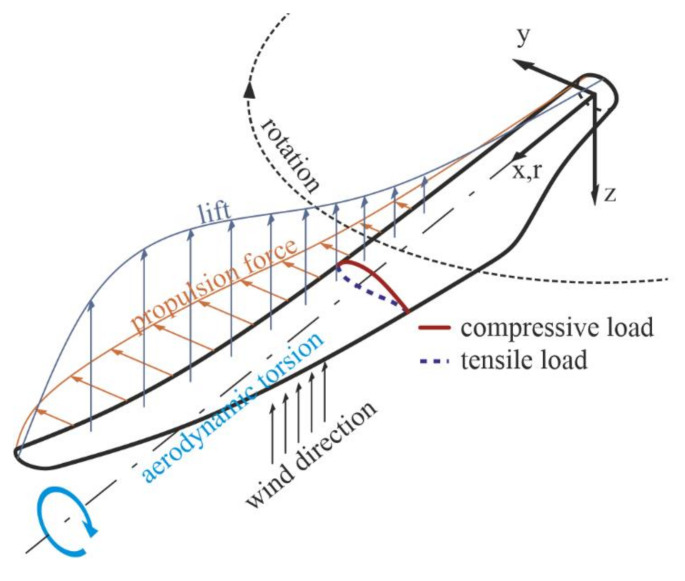
Summary of in-service loads experienced by wind turbine blade shell.

The test specimens were designed to be representative of one section of a scarf repair joint in order to focus specifically on the damage mechanism along this connection between parent and repair patch material and understand the role of the fiber orientation mismatch in the region, highlighted in Figure 2d with a dashed frame. Specimens were produced using the vacuum-assisted resin infusion (VARI) process with biaxial (biax) E-glass fiber non-crimp fabric (NCF) enforced with an epoxy resin matrix. Two different orientations were utilized for the parent material, (0/90°)_2S_ and (±45°)_2S_, which were based on the global loads experienced by an example wind turbine blade with a biax shell layup, illustrated in Figure 3. The out-of-plane impact load from the incoming wind leads to flatwise bending and thereby lateral forces on the blade shell. This in turn makes the ±45° oriented biax shell face sheet laminates undergo tensile and compressive loading. The ±45° specimens in this study are thereby representative of common blade shell structure outer face sheets under tensile and compressive in-service loads. The aerodynamic torsion moment, on the other hand, leads to shear stresses on the blade shell, represented by the 0/90° specimens in this study [16,29,30].

In order to focus on the effect of the fiber orientation mismatch on the damage mechanism of scarf repairs, the scarf slope of the specimens in this study was left constant across all specimens. The scarf ratio was 1:50, corresponding to an angle of 1.15°. This was chosen as a compromise between restored strength and size of patch based on knowledge from prior studies and common practice from FRP repairs in the aerospace and general aviation industry [8,14,16].

**Figure 4:**
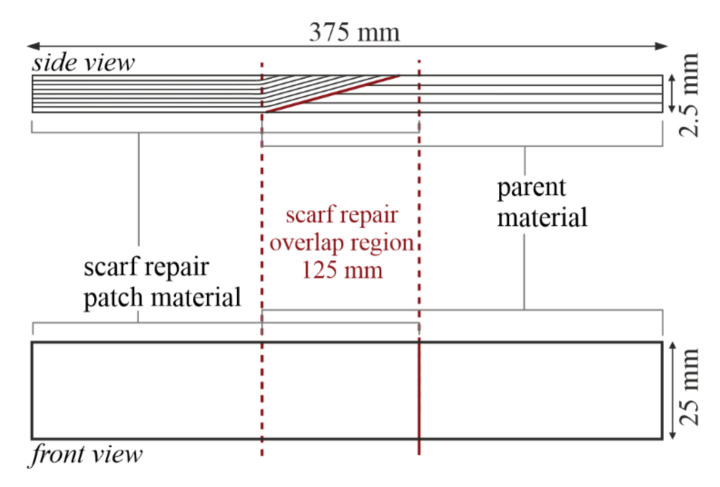
Schematic side (**top**) and front (**bottom**) view of repaired test specimens with scarf joint.

The dimensions of the specimens were designed based on the geometries given in the DIN EN ISO 527-4:1997 Standard for determining the tensile properties of isotropic and orthotropic fiber-reinforced plastic composites [31]. The fiber volume content of the parent material of the specimens was V_f_ = 0.48, while the repair material side was V_f_ = 0.40, determined according to Test Method I of ASTM D 3171-99:2000 [32]. Together with the areal weight and fiber density, this defined the 2.5 mm thickness of the specimens. The width was thus accordingly 25 mm, corresponding to a healthy thickness-to-width ratio of 1:10 and matching the Standard geometry. Lastly, the 375 mm length was designed to be the length of the Standard specimens, 250 mm, plus the additional length of the scarf joint region, which in this case was 125 mm to allow for the 1:50 scarf slope with a laminate thickness of 2.5 mm. The control specimens, although with no scarf joint, were produced with the same dimensions to avoid any geometry effects in the comparison of test results. Figure 4 schematically illustrates the dimensions of the final coupon specimens used in this study, where layers in the side view represent fiber orientations. Thus, two layers of biax GFRP make up four alternating orientations of either ±45° or 0/90°. The repair material features four layers of biax, making eight alternating orientations which correspond to the parent material.

#### 2.1.1. Control Specimens

The specimens were produced as 2.5 mm thick plates using four layers of X-E-778 g/m^2–^1270 mm biax E-glass NCF infused with a pressure gradient of 1 bar with the MGS RIMR135/RIMH137 epoxy laminating resin and hardener system from Hexion (Columbus, OH, USA). The material properties of the resin system are shown in Table 1. Plates were cured at room temperature for 48 h, then post-cured at 80 °C for 15 h. At this stage in the production, reference specimens of full-parent material were cut into the desired specimen size of 25 mm × 375 mm from the plates, while the rest of the plates were further processed to create repaired scarf joint specimens. These full-parent material specimens, taken for comparison and reference to the scarf joint specimens, are further referred to as “control specimens” in this paper.

#### 2.1.2. Repaired Specimens

**Figure 5:**
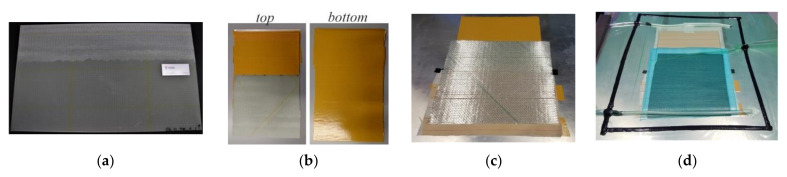
Production process of repaired specimens, as plates. (**a**) Angled surface of parent material prepared for scarf joint to repair layers. (**b**) Top and bottom of parent material plate. (**c**) Repair layer placed onto angled parent material surface for large-to-small layup. (**d**) Vacuum-assisted resin infusion (VARI) setup for infiltration of repair layers with repair epoxy resin system and curing to parent material.

**Figure 6:**
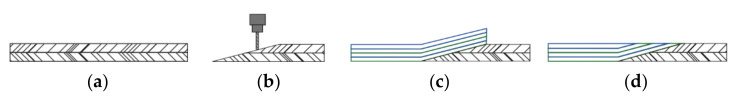
Schematic layup of scarf joint specimens viewed from the side. (**a**) Parent material laminate pre-repair. (**b**) Preparation of repair region with angled removal of laminate with computerized numerical control (CNC) milling machine. (**c**) Layup of new glass fiber-reinforced polymer (GFRP) repair layers onto scarf joint. (**d**) Removal of excess material and surface refurbishing with CNC.

The remainder of the plates produced as reference specimens, described in the previous section, were then further processed to be repaired specimens with a scarf joint. Along the length of the scarf joint, the plates were milled using a computerized numerical control (CNC) machine, always with a milling path at a 45° angle to the fiber orientations in the plate to minimize damage to the fibers, leaving an angled surface for the scarf joint corresponding to a slope of 1:50. After thoroughly cleaning the surface with ethanol, new glass fiber fabric layers were draped with a large-to-small layup, as shown in Figure 5c. The repair side consisted of eight layers of biax X-E-394 g/m^2–^1270 mm E-glass NCF, vacuum-infused with a pressure gradient of 1 bar using the MGS LR285/LH287 epoxy laminating resin and hardener system on a surface heated to 30 °C. The MGS LR285/LH287 system, manufacturing by Hexion (Pernis–Rotterdam, The Netherlands) has a high viscosity and shorter cure time [33], making it highly suitable for repair applications carried out in the field. It is often paired with MGS RIMR135/RIMH137 due to the systems’ similar processing and use temperatures [34]. The material properties of the resin system are shown in Table 1. Repair layers have half the areal weight and are double the amount of the parent material to allow for better drapability onto the fine profile of a flat angled scarf joint while nonetheless being the same laminate thickness. The repair material infusion process onto the angled scarf joint surface is shown in Figure 5, as well as in further detail in Figure 6. All surfaces except the scarf joint were protected from repair side resin infusion by double-sided tape, as shown in Figure 5b. The plates were then left to cure in form for 24 h at room temperature, after which they were post-cured at 80 °C for 15 h.

In order to mimic aerodynamic surface profile restoration performed by sanding repair patches in field repairs, the plate surfaces were then milled back to the original thickness using the CNC machine to remove any excess repair layers, as shown in Figure 6d. biax ±45° GFRP reinforcement tabs with a thickness of 1 mm were applied onto the 0/90° specimens in the 40 mm clamping region. Test coupons were then cut from the bi-material plates into 25 mm × 375 mm coupons, referred to in the remainder of this paper as “repaired specimens.”

**Figure 7:**
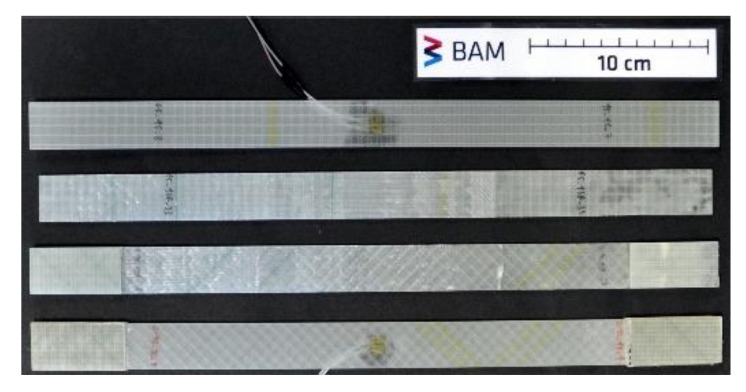
Exemplary specimens of all four types tested in this study, from top to bottom: control ±45°, repaired ±45°, repaired 0/90°, and control 0/90°.

Table 2 outlines the two groups of repaired specimens, with information about the parent layup sequence as well as corresponding repair layers. For each type of repaired specimen, control specimens were also tested for reference for a total of four specimen types. These are depicted in Figure 7.

#### 2.1.3. Mechanical Testing

All specimens were subjected to static tensile load based on the methodology described in DIN EN ISO 527-1:2012 [35] in order to compare the tensile strength and longitudinal stiffness of the control specimens with the scarf joint repaired specimens for both ±45° and 0/90° orientations. A 63 kN capacity Schenk PSB servo-hydraulic testing machine with hydraulic grips was used at a fixed loading rate of 2 mm/min. Strain gauges were used to monitor local strains at the center of the specimen on the parent material side during loading.

### 2.2. Finite Element Analysis

#### 2.2.1. Materials and Properties

Accompanying the experimental testing of scarf joint coupons, a ply-wise stress analysis was conducted with numerical modelling of specimens of the same scarf joint coupon style. The goal of the numerical analysis was to provide insight into the stress distributions affected by a mismatch in fiber orientation, which support the failure mechanisms observed experimentally, especially with respect to inter-fiber failure (IFF) versus fiber failure (FF). Using static-mechanical analysis ANSYS Composite PrepPost (ACP), a tool specifically designated for simulating layup and failure analysis of composite laminates and often used for FRP applications [36,37,38,39], two groups of monolithic scarf joint specimens were modelled to parallel in an idealized manner the structure and geometry of the experimental test coupons.

The material property data for unidirectional (UD) single ply layers of the parent and patch materials, outlined in Table 3 and Table 4, were taken from the literature [40,41]. The elastic stiffness and strength values for the parent and repair material were determined in accordance with DIN EN ISO 527-1:2012 and DIN EN ISO 14126:1999 [35,42].

#### 2.2.2. Geometry

**Figure 8:**
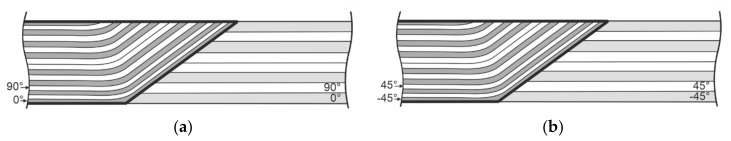
Schematic of two groups of scarf joint specimens, with (**a**) 0/90° biax and (**b**) ±45° biax. Parent layers are shown as the lighter gray region on the right, while repair layers are the darker region on the left.

The specimens in this study were modelled first as the parent material side with eight ply layers corresponding to the four biax layers. The scarf joint region was then removed at an angle corresponding to a scarf ratio of 1:50, and 16 fiber orientations corresponding to the eight biax repair layers were modelled upon this surface for the second half of the specimen. Figure 8 shows a schematic side view of the two fiber orientation specimens in the simulations, where alternating gray/white colors represent alternating fiber orientations.

Second order SOLID186 elements with a quadratic approach function were used in the models. The 12 intermediate nodes ensure lower rigidity of the element and thereby deliver high quality results in the event of major deformations. A convergence analysis showed the difference in the maximum displacement in the longitudinal direction to be significant up to an element size of 10 mm and less than 2% for finer elements. Therefore, a 10 mm element size was determined to be suitable for an independent mesh with results of sufficient accuracy and was adopted in the model. In the scarf joint region, the mesh was refined to 3 mm elements in order to accurately incorporate the more complex geometry, for a total of 53,441 nodes and 11,829 elements in the repaired specimen model.

#### 2.2.3. Boundary Conditions

**Figure 9:**
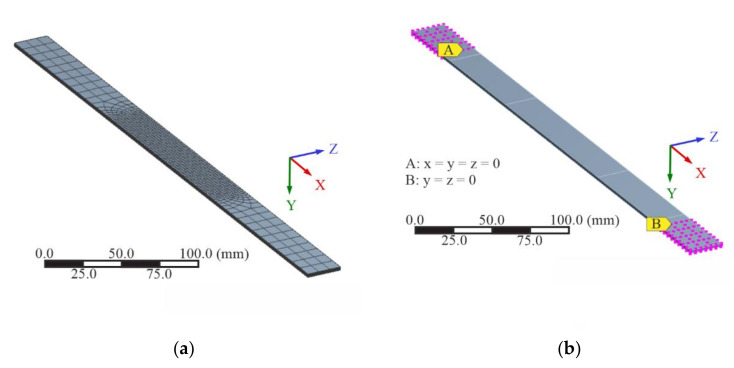
(**a**) Mesh and (**b**) boundary conditions induced upon specimens in the numerical analyses.

The specimens were loaded in the simulations under tension, and the critical regions by layer were compared between the biax 0/90° and ±45° configurations. The displacement applied was corresponding to the experimental results of the static tensile testing, with boundary conditions selected to properly simulate the testing procedure, shown in Figure 9. Nodes in the A region were fixed with x = y = z = 0 mm, representing being clamped and held in place on one end of the test machine. In the B region, the nodes had y = z = 0 mm, while displacement was brought upon the positive x direction, representing the tensile load brought upon by pulling down on one side of the specimen while the other side is held in place.

## 3. Results and Discussion

### 3.1. Tensile Strength and Stiffness of Control and Repaired Specimens

In order to compare the tensile strength and longitudinal stiffness restoration of the specimens with scarf joints, control and repaired specimens were subjected to static tensile load. During testing, the longitudinal strain was measured locally at the specimen center by strain gauges, as well as noted as nominal strain using the percent elongation:

ε_t_ = L_t_/L. (1)

**Figure 10:**
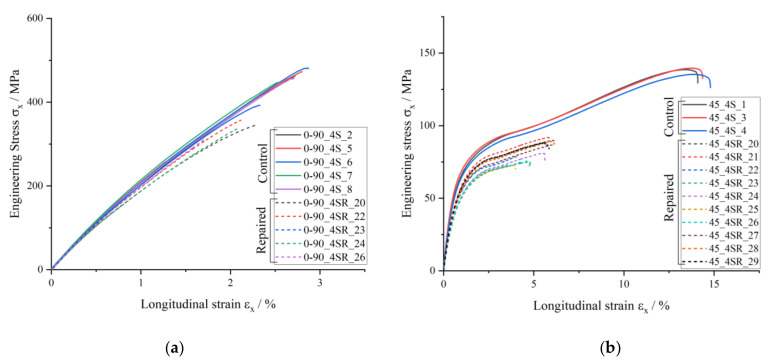
Tensile stress versus longitudinal strain of control and repaired (**a**) 0/90° specimens and (**b**) ±45° specimens. Solid lines show the behavior of the control specimens in both diagrams, while dashed lines are repaired specimens.

Here, ε_t_ is the nominal strain, expressed as a percentage, L is the gripping distance, expressed in millimeters, and L_t_ is the increase in the gripping distance occurring from the beginning of the test [33]. The strain gauges were used to capture the local strain under tensile load at the specimen center, specifically in the scarf joint region. For the purpose of the comparisons between control and repaired specimens in Figure 10, however, the longitudinal strain was given as the nominal strain calculated as percent elongation ε_t_. This was performed in order to not limit the stiffness comparison to a localized point at the center of the specimen where the strain gauge is attached, which is unable to summarize the global deformations due to the complex joint structure. Using the nominal strain allowed for a structural comparison of the global change in stiffness with the introduction of a scarf joint, given the length of the specimens and scarf joint.

The stress–strain curve of the control ±45° specimens in Figure 10b exhibits a second increase in stiffness after a global longitudinal strain ε_x_ of about 6%. This is caused by the fiber rotation occurring due to the load direction compared to the fiber direction, shearing the fibers under tensile load toward the load direction. The angle of rotation can be calculated based on the strain in the longitudinal and transverse directions, expressed in radians [40]:

ω = arctan[(1 + ε_x_)/(1 + ε_y_)] − π/4. (2)

Table 5 below summarizes the rotation (in degrees) at various longitudinal strains, showing a fiber rotation of approximately 6° toward the end of the tensile test. This means that the originally ±45° fibers end up with a ±39° orientation, a hardening effect caused by being slightly stiffer than the original structure and thus having a second stiffness spike in the stress–strain diagram in Figure 10b.

Focusing on the tensile strength of the tested specimens, the average values of both control and repaired were compared and summarized below in Table 6.

In the control 0/90° specimens, the load is carried by a combination of the individual layers in the laminate, namely, the 0° and 90° orientations. Thus, there is a combination of transverse and longitudinal strength of UD GFRP plies at play for this specific sequence. Due to their high stiffness, most of the load is carried by the 0° fibers, which span along the length of the entire specimen in the load direction. This continues until the complete failure of enough 90° layers. Thereby, with the presence of 50% 90° fibers, the ultimate tensile strength ends up being nearly the middle value of the transverse and longitudinal strength of UD 90° and 0° lamina, namely, approximately between 45 MPa and 900 MPa, respectively. This means that a rough estimate value of 430 MPa is expected for the tensile strength of the 0/90° laminate, with just an 8% difference from the 464 MPa outcome from the experimental testing of the control specimens.

With the introduction of a scarf joint in the 0/90° specimens, the experimentally determined tensile strength becomes 341 MPa, 73% of the corresponding reference control 0/90° laminate. As the load was originally carried mostly by the 0° fibers all in the load direction, placing a scarf joint plays a role in the reduction of tensile strength for this fiber orientation. Namely, it inhibits the 0° fibers from carrying the tensile load across the length of the entire specimen. With the presence of a scarf joint, these 0° fibers can only carry the load until the discontinuity in the joint region, leading to failure primarily along the scarf joint at a lower tensile stress than the control specimens. However, the flat angle of the repair joint caused by the 1:50 scarf ratio leads nonetheless to a good load transfer between parent and repair material, thereby achieving a good strength restoration of over 70%.

In the ±45° specimens, there is a similar tensile strength restoration of the repaired specimens, reaching 75%. In these specimens, the flat angle of the 1:50 scarf ratio connecting the two materials also leads to a high-quality joint. This in turn leads to a lack of significant change in failure mechanism caused by the presence of a scarf joint. Both the control and the repaired ±45° specimens under tensile load fail due to intralaminar crack propagation through the specimen thickness, which will be discussed in greater detail in Section 3.3.2.2.

The percent longitudinal stiffness restored was taken as a comparison between the tensile modulus of the repaired versus control specimens of each orientation. The tensile modulus was in turn calculated using the nominal strain $\varepsilon_{t}$ as percent elongation of the specimen length under tensile load, as described above. This was carried out in order to encompass a global effect of the scarf joint in the repaired specimens, referred to as “effective tensile modulus” due to the bi-material nature of these specimens. In both the ±45° and 0/90° orientations, there is a decrease in global longitudinal stiffness in the linear elastic region with the presence of a scarf joint, as presented in Table 7. Noticeable is the significantly higher 96% longitudinal stiffness restoration of the 0/90° repaired specimens in comparison to the 80% restoration of the ±45° repaired specimens. This can be explained by the varying fiber volume contents in the parent versus repair material. A lower V_f_ in the repair material leads to a larger decrease in stiffness in ±45° specimens versus a minimal decrease in stiffness in a 0/90° orientation. In the repaired 0/90° specimens, stiff 0° fibers in the load direction are still present, leading to minimal influence of the matrix. On the contrary, the ±45° repaired specimens under tensile load experience a more matrix-influenced failure, and thus have an 80% stiffness restoration with the presence of a less stiff matrix and lower V_f_ in comparison with the parent material.

### 3.2. FEA Validation

The FEA allowed for an understanding of the distribution of critical regions of a structure with a scarf joint, depending on layup configuration, under tensile load. To test the predictive capability of the scarf joint specimen simulations, a comparison of the load and strain between the experimental and numerical model tensile results for the control specimens was conducted. Since the 0/90° control specimens experience a slight stiffness degradation caused by an increase in IFF, the values at the end of the linear elastic region were compared with the simulation values in Table 8. The end of the linear elastic region was calculated using the 0.2% offset yield point procedure on the stress–strain diagram to be at a displacement of 3.44 mm. The difference in the 0/90° orientation FEA values in comparison to the experimental ones can be explained partly by the idealized nature of the numerical specimens, which are free from all manufacturing errors, and can be partially due to the adoption material properties of E-glass fiber values from the literature.

**Figure 11:**
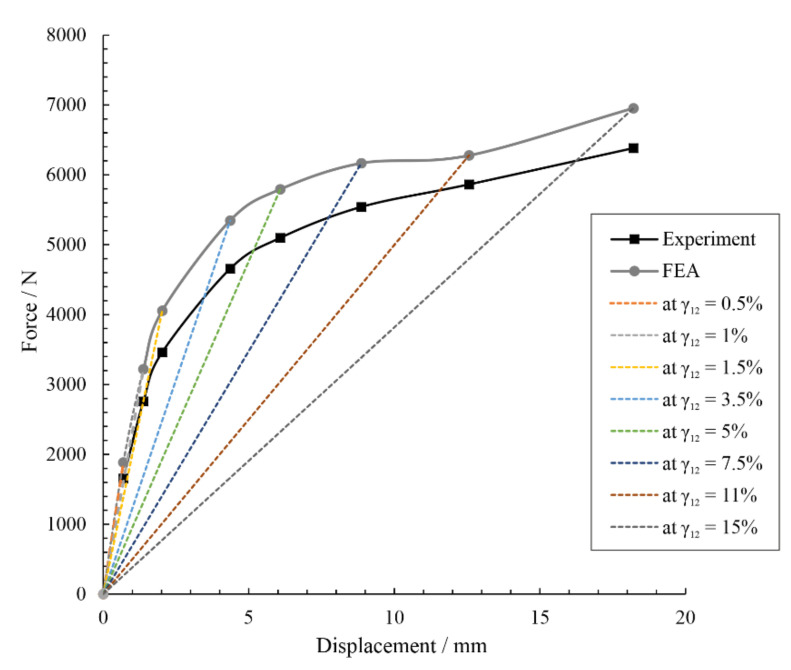
Incremental comparison of experimental and numerical control ±45° specimens under tensile load with calculated shear modulus values at each step fed into the multilinear material model for the finite element simulations.

For the ±45° control specimens, which experience significant plastic deformation before fracture until tensile load, shear modulus values were calculated using the inverse laminate theory for various points along the applied load curve, and the simulations were run step-wise with these calculated shear modulus values at each point. These values are outlined in Table 9. The resulting load and displacement curves were compared between experimental and numerical specimens, shown in Figure 11. These step-wise shear modulus values were then utilized in the numerical analyses of the repaired ±45° specimens, elaborated in Section 3.3.2.2.

There was good agreement between the ±45° experimental and numerical specimens’ performance. The slight percent difference (between 7–17%, depending on load step and orientation) is attributed partially to the idealized nature of the numerical specimens in comparison with the experimental ones with composites manufacturing nuances. Additionally, the crack formation during experimental tensile testing of the ±45° specimens leads to a tendency toward non-linearity, also contributing to differences in the finite element (FE) model values. The FE model was shown to nonetheless be sufficient in providing insights into stress concentrations that abet the failure processes highlighted by the experimental study.

### 3.3. Failure Mechanisms

The method of damage initiation and propagation in the scarf joint repaired specimens of both orientations was examined experimentally, while finite element analysis gave insights into stress distributions in the structure using a layer-wise stress analysis. In this section, experimental results are presented with insight from numerical simulation results.

For the purpose of the numerical analysis of this study, the failure criteria according to Puck was applied due to its enveloping nature as well as ability to differentiate between various failure modes. The stresses which were calculated for each ply were compared to the Puck failure criteria to determine the inverse reserve factor (IRF) and thereby identify the critical regions, elaborated in the following section. These criteria utilized in the ACP Post environmental consider failure under numerous loading methods: fiber failure due to longitudinal loading, as well as IFF under transverse and shear loading [43,44]. They were therefore used in this study to determine the critical positions in the scarf joint laminates subject to tensile load. The next section details the theory behind the Puck failure criteria applied with use of the IRF, while the following sections present the numerical results in the context of the experimental results.

#### 3.3.1. The Inverse Reserve Factor

For the numerical simulations in this study, the modified Puck failure criteria were used in ANSYS to produce IRF values as a metric for determining critical regions in the specimens. The parameters used to define the failure functions were taken as typical GFRP values in ANSYS, summarized in Table 10. $p_{⊥∥}^{\left(+\right)}$ and $p_{⊥∥}^{\left(-\right)}$ are the slopes of the $\left(\sigma_{2},\;\tau_{21}\right)$ inter-fiber failure curve where $\sigma_{2}=0$.

To compare the numerical results of the repaired specimens, the IRF was used. The reserve factor (RF) of a structure describes its margin to failure and is defined as

RF = σ_failure_/σ_applied_. (3)

An RF > 1 indicates a positive margin to failure. Thus, the critical values lie between zero and one, whereas non-critical values range from one to infinity. As the non-critical values are often emphasized, the IRF is commonly used, where the non-critical values range from zero to one, where

IRF = 1/RF.(4)

Corresponding to the Puck fracture curve of plane states of stress, the IRF is described as the stress exposure, f_E_:

f_E_ = {σ}/{σ}_fr_. (5)

The stress exposure is a ratio of the length of the actual stress vector applied on the body {σ} over the length of the vector of the stresses that lead to fracture {σ}_fr_ [42,43,44,45,46].

#### 3.3.2. Damage Initiation and Propagation

Damage initiation and propagation for the repaired specimens with the 0/90° and ±45° orientation varied based on the failure mechanisms at play, discussed in greater detail in the following sections.

##### 3.3.2.1. 0/90°. Repaired Specimens

**Figure 12:**
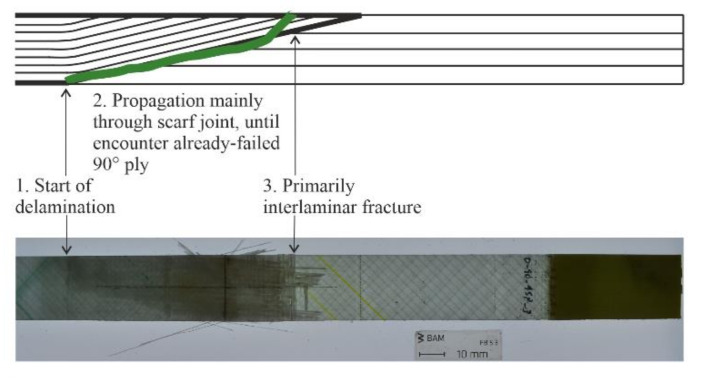
Step-by-step fracture path of 0/90° repaired specimens under tensile load, portrayed as the top view of a real tested specimen (**bottom**) and side view of a schematic illustration (**top**).

The damage initiation for the 0/90° repaired specimens began at the edge of the scarf joint, on the side with more repair material, shown in Figure 12. At this point is a resin fillet caused by a slight jump (0.1 mm) in material caused by the joining of the repair material with the original parent structure. As opposed to the other side of the scarf joint, where there is more parent material, this side’s surface profile is not restored after repair layers are applied. This side is representative of the back side of a wind turbine blade shell face sheet which is repaired, the side in contact with the core material of the sandwich structure, illustrated schematically in Figure 18 of Section 4, and plays a more significant role in the failure of the 0/90° repaired specimens due to the role of the 0° layers influencing the specimen strength and stiffness.

After failure initiates at this point, there is a competition of interlaminar strength versus fiber strength at play when it comes to the propagation path. Unlike the control 0/90° specimens, there are no longer continuous 0° fibers running along the entire specimen length in the load direction that are able to carry the load. Here, the interlaminar strength is significantly lower than the fiber strength in the load direction, leading to interlaminar propagation of the failure path along the scarf joint, shown in Figure 12. Additionally, the peak shear stresses in the structure are lower with the presence of 0° plies [17]. This propagation in the interface between parent and repair material continues in this way until an already failed 90° layer in the repair is reached, after which the failure propagates for the last small portion toward the surface of the specimen through the repair material.

**Figure 13:**
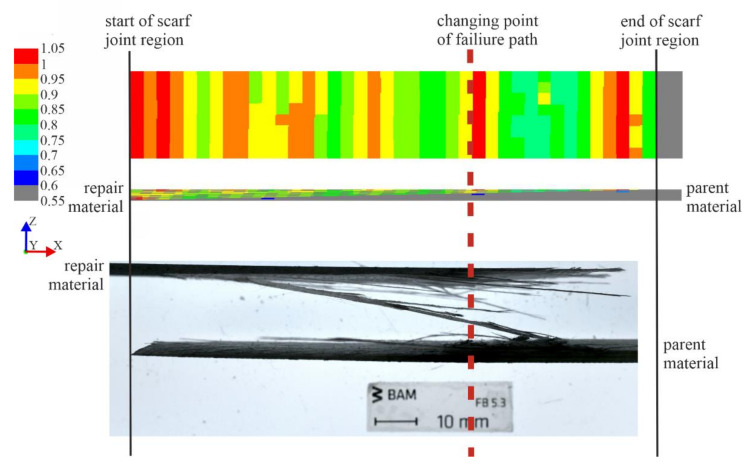
Critical regions of numerical 0/90° scarf joint specimen under tensile load at σ_x_ = 48 MPa (occurrence of first inter-fiber failure (IFF)) presented as inverse reserve factor (IRF) values projected onto the specimen front and side view. In comparison, the side view of the two fracture surfaces of an experimental 0/90° scarf joint specimen tested under tensile load is shown. The scale begins at a value of 0.55 for clarity.

The change of the failure path from being interlaminar through the scarf joint to propagating for the last portion through the repair material is compared with the IRF projection of a repaired 0/90° specimen in Figure 13. Using the Puck failure criteria, the numerical model was used to determine the critical regions with a value greater than 1 in each ply, namely, regions which have undergone IFF, which in this case was Mode A. This type of failure is especially interesting, as the IFF disrupt the force flux, which can create interlaminar shear stresses. The 3D stress state promotes delamination in these areas [44]. The IRF values of each of the plies in the model were projected onto a fictional two-dimensional plane to visualize the overall distribution of critical regions in the scarf joint region, as if viewing the test specimen from the top (as in Figure 13, top) or side (as in Figure 13, middle). Figure 13 thus compares the IRF of a 0/90° repaired specimen under tensile load with the side-view geometry of the same specimen that has failed experimentally. The IRF values in this image are taken at the first occurrence of IFF at a displacement of 0.63 mm and a stress of σ_x_ = 48 MPa.

The side and front view of the IRF values in Figure 13 show the red critical regions (IRF value greater than 1) in the specimen within the scarf joint region, and can be mapped to the experimental damage initiation site as well as point at which the propagation path changes from interlaminar in the scarf joint to intralaminar through the repair material. Here, the fracture front, carrying the highest energy at the crack tip, encounters an already-failed 90° ply layer, namely, the first instance that a critical region comes into contact with the scarf joint interface after initiation. At this point, the failure mechanisms at play change from fiber failure versus interlaminar failure to fiber failure versus interlaminar failure versus the new option introduced by the failed 90° layer in contact with the scarf joint interface: intralaminar failure. The high-energy fracture tip then changes its direction to propagate for the last portion through the remaining repair material layers to the specimen surface until final failure. This is also noticeable in the abrupt change from smooth fracture surface between the two materials to repair material fibers that are partially still intertwined in the side view of the fracture surface of the experimental specimen in Figure 13. The critical regions in the model align to the point at which failure initiates in the experimental specimen, as well as when the failure path changes from interlaminar to intralaminar for the final propagation through the repair material to the specimen surface.

From this comparison, it can be concluded that the failure path remains interlaminar until an already-failed 90° ply, namely, a region with a critical IRF value, comes in contact with the scarf joint interface. This occurs at the point marked in a red line in Figure 13. This type of fracture path change behavior has also been observed in the literature, where failure in scarf repaired CFRP specimens under tensile load occurred as cracks initiating either outside the scarf joint bond line or possibly at the bond line contacting a 90° ply, and the propagation occurred as a staircase path, changing direction into the laminate when in contact with an already-failed 90° ply. This could be explained by the effect of the interfacial joint stiffness being indifferent from that of the parent and repair substrates, thereby conveying some of the scarf joint interface strain behavior to a compliant 90° ply and including this ply as part of the joint line. The 90° ply is then forced to adopt the shear strain behavior of a ductile adhesive, undergoing a shear distortion at a magnitude on the order of that along the bond line, ultimately leading to cracks in the 90° layer [11]. This type of stepped crack path changing behavior at the bond line due to 90° plies was also observed in the literature [3,11].

##### 3.3.2.2. ±45°. Repaired Specimens

On the contrary to 0/90°, the mechanisms at play in the repaired ±45° specimens under tensile load are interlaminar strength and intralaminar strength. If the interlaminar bond between the newly brought upon repair layers and original parent structure is stronger than the intralaminar bond within each ply, the fracture strength should be minimally affected by the presence of the scarf joint, as is the case with the 75% tensile strength restoration discussed in Section 3.1. The load is not carried by fibers that extend along the entire length of the specimen in the load direction, as is performed by the 0° plies in the 0/90° specimens. Free edge effects in the specimens further support the favoring of intralaminar failure. Thus, the means in which the load is carried in the ±45° repaired structure is mostly unaffected by the presence of a scarf joint.

**Figure 14:**
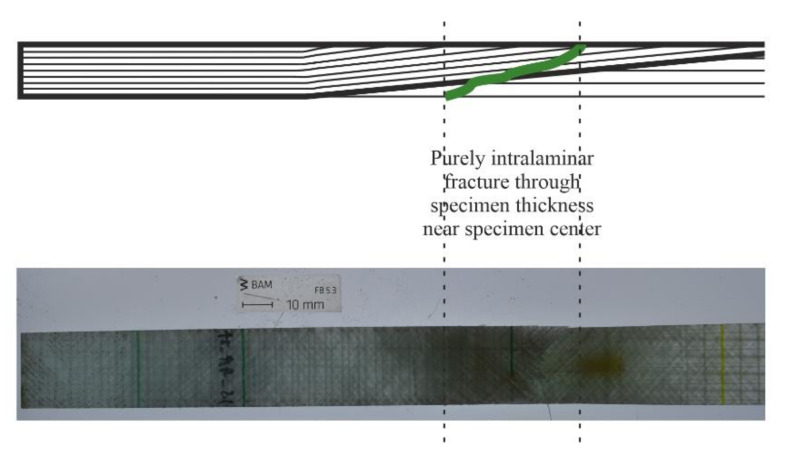
Fracture path of ±45° repaired specimens under tensile load, portrayed as a top view of a real fractured specimen (**bottom**) and the side view of a schematic illustration (**top**).

After initiation at the specimen edges, the damage propagation path of the repaired ±45° specimens is through the specimen thickness, as is the case for the control specimens of the same orientation. This is visualized in Figure 14, which shows the failure path of a repaired ±45° specimen. The repaired ±45° specimens also underwent a period of localized necking before failure, which has also been observed in ±45° FRP tensile testing of specimens without scarf joints. Video recording of the tests showed that the necking occurs near the center of the specimen, in the region in which fracture occurs, beginning at approximately ε_x_ = 2.3–3% strain and σ_x_ = 70–80 MPa stress. This is likely due to the concentration of IFF already having occurred in this region, making the material more elastic and thereby undergoing more local deformation. This pliable local strain leads to a high increase in longitudinal strain until fracture at an average ε_x,max_ = 5–6%, nearly double that at the start of the necking. Contrastingly, the ultimate tensile strength of the repaired ±45° specimens is σ_x,max_ = 80–90 MPa, which is only less than 15% greater than the stress at necking onset.

**Figure 15:**
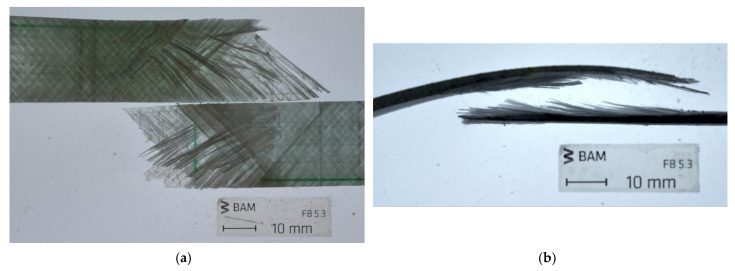
(**a**) Fractured surfaces and (**b**) side view of ±45° scarf joint specimen under tensile load, showing fibrous remnants as a result of the intralaminar fracture path.

Compared to the smooth surface showing interlaminar failure along the scarf joint in the 0/90° specimens, the ±45° repaired specimens have a fibrous fracture surface concentrated in the region of failure, shown in Figure 15. This is a result of the unchanged intralaminar failure path through the specimen thickness, as occurs in the control specimens.

**Figure 16:**
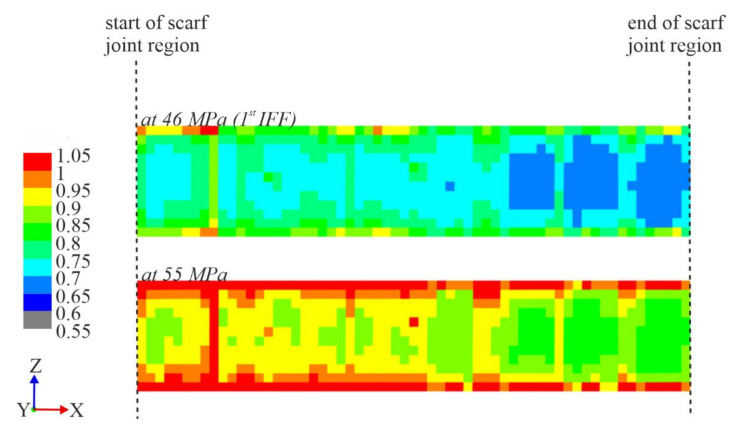
IRF values of ±45° scarf joint region, showing the development of the critical regions under tensile load, viewed from the specimen top. The scale begins at a value of 0.55 for clarity.

**Figure 17:**
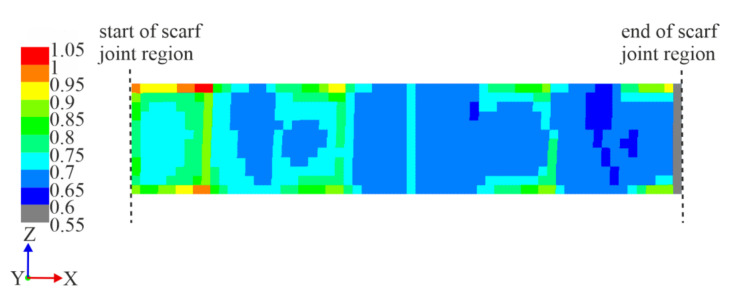
IRF values of the bottom-most repair layer of a simulated ±45° scarf joint specimen under tensile load from the top view, shown to be the most critical layer under tensile load, where the first IFF occurs at σ_x_ = 46 MPa. The scale begins at a value of 0.55 for clarity.

The numerical model of the repaired ±45° specimens were utilized for insight into more detail to the failure mechanism. The analyses were conducted using the modified step-wise shear modulus values calibrated from the experimental results of the control ±45° specimens, described in detail in Section 3.2. As with the 0/90° specimens, the IRF was calculated ply-wise, then projected onto a fictional two-dimensional plane for a visualization of the distribution of the critical regions within the scarf joint region and to identify the ply in which failure first occurs. Figure 16 shows the projected IRF values within the scarf joint region at a longitudinal stress of σ_x_ = 46 MPa, where the first IFF occurred (top) and at a later stress of σ_x_ = 55 MPa to compare the development of the critical regions (bottom). The first IFF at σ_x_ = 46 MPa was shown to be the bottom-most +45° repair layer, whose single layer IRF distribution is shown in Figure 17.

Looking at the IRF values of the scarf joint specimens in the ±45° orientation in Figure 16, it can be seen that the critical regions were concentrated mostly along the edges of the specimen, as is the case for control ±45° specimens under tensile load. In contrast to the 0/90° repaired specimen, which showed higher IRF values in the repair layers than parent material, and especially at the points which mapped to damage initiation and damage path changes, the ±45° stress exposure seems uninfluenced by the presence of a scarf joint, thereby confirming the efficiency of the scarf joint. Therefore, the failure path of the repaired specimen remains intralaminar as in the control ±45° coupons.

## 4. Conclusions

**Figure 18:**
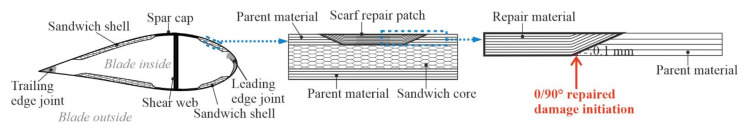
Hierarchical representation of scarf joint coupon specimens in this study (**far right**), as monolithic face sheet of a sandwich structure (**middle**) found in the shell of blades, shown in the blade cross-section (**far left**). The coupon image (**far right**) depicts the damage initiation location of the repaired 0/90° specimens.

The performance of scarf joint GFRP specimens under tensile load was compared in this study both experimentally as well as numerically for two different groups, corresponding to fiber orientations with respect to in-service loads experienced on biax wind turbine blade shells. Based on the work carried out, the following conclusions can be summarized:

- There was an overall good ultimate tensile strength restoration in both orientations of over 70%.
- The matrix properties and V_f_ of the repair material played a larger role in the stiffness restoration of the ±45° repaired specimens (versus 0/90° repaired) due to the greater influence of the matrix in the failure under tensile load.
- The restoration of the surface profile after joining the repair layers played a significant role in influencing the damage initiation. The surface of specimens in this study milled with the CNC machine were representative of the outside of the blade shell, which is sanded after repair to restore the aerodynamic profile. The back side of the repair, the side in contact with the core material and in the case of this study the side with more parent material, is left as-is, a minimal 0.1 mm resin fillet. This results in damage initiation in the 0/90° repaired specimens as delamination at this point shortly after tensile load is applied, shown in bold in Figure 18.

- The failure mechanism varies based on orientation because of the mechanisms at play under tensile load. The crack propagation path always takes the direction of least resistance.
- The failure mechanism of ±45° repaired specimens remained the same as the control specimens, as intralaminar strength is less than interlaminar strength. The failure path therefore follows intralaminar propagation through specimen thickness, with no influence of scarf joint on failure path.
- The failure mechanism of 0/90° specimens changes with the presence of a scarf joint to failing between the repair and parent material, along the joint. This is because here, with the presence of 0° fibers in the load direction, the mechanisms at play are fiber strength versus interlaminar strength, the weaker of the two being interlaminar and therefore seeing the propagation path mainly along the scarf joint.
- In the repaired 0/90° specimens, when an already-failed 90° layer in contact with the joint interface is encountered, the mechanisms at play change again to being interlaminar versus intralaminar strength, by which the failure path then changes to intralaminar failure through the repair material to the specimen surface.
- The 73% ultimate tensile strength restoration despite the change of failure mechanism and fiber orientation mismatch of the repaired 0/90° specimens speaks for the effectiveness and quality of a 1:50 scarf joint.

These findings can be applicable to and enhance good repair design, especially in the wind turbine blade industry, as the components experience a range of in-service loads which can differently affect repair patches.

## Figures and Tables

**Table 1:** Material properties of the RIMR135/RIMH137 and LR285/LH287 epoxy resin and hardener systems [33,34].

Resin System	Density/g/cm^3^	Modulus of Elasticity/GPa	Elongation at Break/%	Tensile Strength/MPa
RIMR135/RIMH137	1.18–1.20	2.7–3.2	8–16	60–75
LR285/LH287	1.18–1.20	3.0–3.3	5.0–6.5	70–80

**Table 2:** Layup configurations of numerical simulation specimens modeled with ANSYS and tested experimentally under static tensile load.

Group Name	Parent Sequence	Parent Materials	Repair Sequence	Repair Materials
biax ±45°	[−45/+45]_2S_	2 × 778 g/m^2^ biax E-glass NCF, RIMR135/RIMH137 epoxy	[−45/+45]_4S_	4 × 394 g/m^2^ biax E-glass NCF, LR285/LH287 epoxy
biax 0/90°	[0/90]_2S_	2 × 778 g/m^2^ bax E-glass NCF, RIMR135/RIMH137 epoxy	[0/90]_4S_	4 × 394 g/m^2^ biax E-glass NCF, LR285/LH287 epoxy

**Table 3:** Parent material property data used in using finite element analysis (FEA) of control and repaired specimens [41].

E_∥_/MPa	E_⊥_/MPa	G_⊥∥_/MPa	ν_∥__⊥_	ν_⊥__∥_
41,208	9628	3539	0.1	0.3
$R_{∥}^{\left(+\right)}$_/_MPa	$R_{∥}^{\left(-\right)}$_/_MPa	$R_{⊥}^{\left(+\right)}$_/_MPa	$R_{⊥}^{\left(-\right)}$_/_MPa	R_⊥__∥/_MPa
865	−715.2	40.6	−124.8	41.3

**Table 4:** Repair material property data used in FEA of repaired specimens [40].

E_∥_/MPa	E_⊥_/MPa	G_⊥∥_/MPa	ν_∥⊥_	ν_⊥∥_
32,380	8990	2830	0.1	0.3
$R_{∥}^{\left(+\right)}$_/_MPa	$R_{∥}^{\left(-\right)}$_/_MPa	$R_{⊥}^{\left(+\right)}$_/_MPa	$R_{⊥}^{\left(-\right)}$_/_MPa	R_⊥__∥/_MPa
865.4	−541.6	23	−107.5	49.2

**Table 5:** Control ±45° specimen fiber rotation at various points during static tensile test.

Longitudinal Strain ε_x_/%	Transverse Strain ε_y_/%	Fiber Rotation ω/°
0.2	−0.09	0.1
3.0	−1.7	1.3
5.0	−2.9	2.3
10.0	−62	4.5
11.0	−9.9	6.0

**Table 6:** Comparison of tensile strength restoration between control and repaired specimens, experimental results.

	Tensile Strength—Control/MPa	Tensile Strength—Repaired/MPa	Tensile Strength Restored/%
biax 0/90°	464	341	73
biax ±45°	110	83	75

**Table 7:** Comparison of longitudinal stiffness restoration between control and repaired specimens, experimental results.

	Tensile Modulus—Control/GPa	Effective Tensile Modulus—Repaired/GPa	Longitudinal Stiffness Restored/%
biax 0/90°	23.2	22.2	96
biax ±45°	10.0	8.0	80

**Table 8:** Comparison of experimental and numerical results of tensile test for control 0/90° specimens taken at 3.44 mm displacement, the calculated end of the linear elastic region.

	Experiment	Simulation	Error
Longitudinal strain ε_x_/%	1.09	1.03	5.82%
Load/kN	14.5	18.1	19.89%

**Table 9:** Step-wise calculated shear modulus G_12_ values for control ±45° specimens, used for multilinear verification of numerical model.

Step-Wise Shear Strain γ_12_/%	Shear Stress τ_12_/MPa	Displacement/mm	Step-Wise Calculated Shear Modulus G_12_/MPa
0.5	14	0.69	2737
1	23	1.38	2260
1.5	28	2.02	1889
3.5	38	4.36	1090
5	42	6.08	833
7.5	45	8.86	604
11	48	12.57	435
15	52	18.21	345

**Table 10:** Puck failure criteria parameters used in the FE model **[46]**.

$p_{⊥∥}^{\left(+\right)}$	$p_{⊥∥}^{\left(-\right)}$	$p_{⊥⊥}^{\left(+\right)}$	$p_{⊥⊥}^{\left(-\right)}$
0.3	0.25	0.2	0.2

## Data Availability

The raw/processed data required to reproduce these findings cannot be shared at this time as the data also form part of an ongoing study.
